# Anaplastic Thyroid Cancer: A Review of Epidemiology, Pathogenesis, and Treatment

**DOI:** 10.1155/2011/542358

**Published:** 2011-06-12

**Authors:** Govardhanan Nagaiah, Akm Hossain, Colin J. Mooney, James Parmentier, Scot C. Remick

**Affiliations:** ^1^Mary Babb Randolph Cancer Center, West Virginia University, Morgantown, WV 26508, USA; ^2^Ellis Fischel Cancer Center, University of Missouri, Columbia, MO 65203, USA; ^3^Department of Medicine, University of Michigan, Ann Arbor, MI 48109, USA; ^4^Graduate Program in Clinical Investigation, MGH Institute of Health Professions, 36 1st Avenue, Charlestown Navy Yard, Boston, MA 02129, USA

## Abstract

Anaplastic thyroid cancer (ATC) is an uncommon malignancy of the thyroid. Only 1-2% of thyroid cancers are anaplastic, but the disease contributes to 14–50% of the mortality with a median survival of 3 to 5 months. Most patients diagnosed with this disease are 65 years of age or older. The incidence of anaplastic thyroid cancer is decreasing worldwide. Most patients present with a rapidly growing neck mass, dysphagia, or voice change. We performed a comprehensive literature search using PubMed focusing on the treatment of anaplastic thyroid cancer including historical review of treatment and outcomes and investigations of new agents and approaches. A total of sixteen chart review and retrospective studies and eleven prospective studies and/or clinical trials were reviewed. The current standard therapeutic approach is to consider the disease as systemic at time of diagnosis and pursue combined modality therapy incorporating cytoreductive surgical resection where feasible and/or chemoradiation either concurrently or sequentially. Doxorubicin is the most commonly used agent, with a response rate of 22%. Several new agents are currently under investigation. Referral of patients for participation in clinical trials is needed.

## 1. Introduction

Anaplastic thyroid cancer (ATC) is one of the most aggressive solid tumors to affect humans, with a median survival on the order of 3 to 5 months following diagnosis [1–25]. One-year and 10-year survival rates are estimated at 10–20% and less than 5%, respectively, though some reports question the reliability of diagnoses in these long-term survivors [11, 26, 27]. Even though less than 1–3% of all thyroid cancers are anaplastic tumors, it contributes up to 14–50% of the annual mortality associated with thyroid cancer [8, 11, 28]. Recent advances in understanding the genetic and molecular pathogenesis of ATC hold promise for targeted therapy for this disease. ATC is usually resistant to standard chemotherapy. There are several clinical trials underway with small molecule tyrosine kinase inhibitors, antiangiogenesis agents and vascular-disrupting agents which might offer more drugs in the therapeutic armamentarium to combat this disease. 

## 2. Clinical Experiences at Our Institution

We identified all the cases of ATC from 1987 to 2007 at the Mary Babb Randolph Cancer Center, Morgantown, WVa, USA. Appropriate IRB approval was obtained. Data was collected on demographics, grade, stage, and different modalities of treatment and survival. SPSS was used to analyze the demographic and survival data.

 A total of 11 cases were identified at our cancer center from 1987 to 2007. Out of these patients, 7 (64%) were female and 4 (36%) were male (*P* < .001). The average age of diagnosis was 69 years. The average age was 70 years among female and 67 years among male patients. All of these patients were white and had grade IV disease (100%) at diagnosis. Eight (73%) patients had stage IV diseases at the time of diagnosis, and only three patients (one from each stage) had stage I to III diseases at the time of diagnosis. Most patients received combination therapy that consisted of surgery and radiation. Eight (73%) had surgery, eight (73%) patients received radiation therapy, and only 5 (45%) patients received chemotherapy. Patients with stage IV diseases at diagnosis, who received surgery followed by radiation therapy, had significantly longer survival than the patients with stage 4 diseases at diagnosis who received surgery after chemotherapy or radiation therapy (44 months versus 9 months, *P* < .0001). The patients who received surgery had significantly better survival than the patients who did not receive surgery (*P* < .001). In our cohort of 11 patients, multimodality treatment consisting of surgery in combination with either radiation or chemotherapy had better outcomes than any of these treatments alone.

## 3. Incidence

ATC is primarily a disease of the elderly. In one American and German prospective study of 5,583 cases of thyroid carcinoma, 67% of patients who had ATC were over 70 years of age. In the same study, females constituted 70% and males 30% of ATC patients [7]. 

Based on epidemiological studies derived from the Surveillance, Epidemiology and End Results (SEER) database, the incidence of ATC has held steady in the United States during the period between 1973 and 2002 [29]. The incidence of ATC is estimated at 1 to 2 cases per million population per year, and the trend has been decreasing even though the incidence of well-differentiated subtypes (e.g., papillary and follicular) of thyroid cancer has been increasing [29]. In a retrospective cohort analysis done by Davies and colleagues using the SEER database, there were roughly 24,000 new cases of thyroid cancer in 2002 of which ATC constituted 2% [29]. A study done in Rochester, Minnesota found that the age-adjusted incidence of ATC was 0.1 per 100,000 person/year (95% Confidence Interval (CI) 0.0–0.3) [2]. From 1973 to 2002, there was a 2.4-fold increase in all thyroid cancer with no significant change in the incidence of ATC. Papillary thyroid cancer, which increased from 2.7 to 7.7 per 100,000, contributed to almost all of the increase. It should be noted that cancers less than one centimeter accounted for 49% and tumors less than two centimeter accounted for 87% of the increase in the period between 1988 and 2002. There was no change in the mortality associated with thyroid cancer during the same period. This raises the question whether early diagnosis was leading to unnecessary interventions. 

 Studies published from Italy showed a reduction of ATC from 4% to 1% between 1969 and 1973, while another study in India showed a decline from 8% to 4% between 1989 and 1993 [42–44]. Although well-differentiated thyroid cancer has increased in Japan, the incidence of ATC has remained stable [45, 46]. In Sri Lanka, a study that looked at the incidence of thyroid cancer over 28 years, reported a significant reduction of anaplastic histology [47]. The incidence of ATC did not change significantly in a study from Scotland [48]. One for the decreasing incidence of ATC in these several locations could be that a more accurate diagnosis is being made. 

## 4. Risk Factors

In one study, 25% of the ATC patients had a prior history of thyroid goiter and another 10% with family history of goiter [7]. Other studies have reported a similar association between goiter and ATC [49]. It is also known that ATC is more common in places with endemic goiter, and thus with improvements in iodine supplementation, the incidence of this tumor would be expected to decline. Besic and colleagues report in a study from Slovenia that when the iodination of salt was increased from 10 mg of potassium iodide/kg since 1972 to 25 mg of potassium iodide/kg between 1997 and 1998 to 2008, the mean incidence of ATC decreased from 6.2 (range, 3–12) between 1972 and 1997 to 4 (range, 2–10) between 1998 and 2008 [50]. 

## 5. Presentation

Local symptoms of ATC most commonly begin with a rapidly evolving central neck mass (77%) followed by noticeable dysphagia (40%), voice change or hoarseness (40%), and stridor (24%). Regional symptoms included a noticeable lymph node mass (54%) and neck pain (26%). Systemic symptoms include anorexia, weight loss, and shortness of breath with pulmonary metastases. Metastases were found in 50% of patients at presentation with 25% developing metastasis during the course of disease. Lungs (80%), bone (6–16%), and brain (5–13%) were the most common sites of metastasis [8]. 

## 6. Prognosis

In an analysis of survival of ATC patients from the SEER database from 1983 to 2002, which included patients who survived for more than a month, the median survival was 4 months. On multivariate analysis, distant or metastatic disease, tumor size greater than 7 centimeters, and treatment with surgery with or without radiotherapy were statistically significant prognostic markers (*P* ≤ .05). Of interest, when the authors stratified patients by the extent of disease and looked into the benefit of radiotherapy after surgery, they found that patients with extracapsular extension into adjacent tissue, the addition of radiotherapy was of benefit. Radiotherapy after surgery was of no benefit in patients who had disease confined to the thyroid or had distant metastasis [51]. Age, sex, size of the tumor, resectability, and the extent of disease has been shown to affect the course of the disease [27]. In a SEER-based study in the United States by multivariate analysis, only age less than 60 years, an intrathyroidal tumor, and the combined use of surgical and external beam radiation therapy were identified as independent predictors of lower cause-specific mortality [52]. In other series, female sex, tumor size less than 6 centimeters, age, and the extent of disease were the most favorable prognostic markers [23, 53]. Among Koreans less than 60 years of age, tumor size less than 7 cm, and lesser disease burden were independent predictors of lower mortality [54]. A recent study from France based on 26 patients with ATC, univariate analysis showed that age above 75, capsular invasion, lymph nodes metastasis, tumor residue after surgery, and lack of multimodal treatment (particularly radiotherapy in patients without tumor residue) are poor prognosistic factors. Multivariate analysis in the same cohort showed age above 75, followed by node invasion, capsular invasion, and female sex to be poor prognosticators [55]. In a study by Venkatesh and colleagues, patients with localized disease had a median survival of 8 months in comparison to 3 months for patients with metastatic disease [24]. A prognostic index was developed by Sugitani and colleagues from a review of their series of 47 patients over 33 years [56]. Their index was based on a combination of four risk factors: (1) presence of acute symptoms, (2) tumor size greater than 5 centimeters, (3) distant metastasis, and (4) white cell count ≥ 10,000/*μ*L [56]. Patients with a prognostic index less than or equal to one had a 62% survival rate at 6 months, whereas all patients with prognostic index of three and four died within six and three months, respectively. 

## 7. General Therapeutic Approach

Patients with ATC even in the absence of metastatic disease are considered to have systemic disease at the time of diagnosis. All ATCs are considered stage IV by the International Union Against Cancer (UICC)—TNM staging and American Joint Commission on Cancer (AJCC) system. Multimodality treatment consisting of surgery when feasible combined with radiation and chemotherapy is generally recommended. The surgery must not compromise the functional anatomy of the cervical structures. The most common cause of death is invasion of vital local structures in the neck. Consequently, achieving good local control with surgery and or radiation confers short term palliative and survival benefit. Radiotherapy alone and in combination with surgery has been shown to achieve good local control in many studies. There is no consensus on the sequence of radiation and surgery. A summary of retrospective and prospective studies with the most important results are presented in Tables 1 and 2, respectively.

## 8. Radiation

Radiation does not alter the course of ATC in most patients. On the other hand, when combined with surgery and chemotherapy, it can prolong the short-term survival in select and subset of patients. Intensity-modulated radiation therapy (IMRT) based on computerized treatment planning and delivery is able to generate a dose distribution that delivers radiation accurately with sparing of the surrounding normal tissue [57, 58]. Higher doses of radiation can be given over a shorter time with less toxicity by employing hyperfractionation techniques [59, 60]. Toxicity can be a limiting factor with radiation. Kim and Leeper reported complications particularly, pharyngoesophagitis and tracheitis in their series [12]. Wong and coworkers also noted skin changes, esophageal toxicity, and radiation myelopathy [59]. Daily doses of greater than 3 Gy should be cautiously used as it can increase the incidence of myelopathy [59]. 

A report from MD Anderson Cancer Center compared outcomes for ATC patients treated with conformal 3-dimensional radiotherapy (3DRT) or IMRT. Of the 53 consecutive patients were analyzed, 31 patients were irradiated with curative intent with a median radiation dose of 55 Gray (Gy; range, 4–70 Gy). Thirteen patients received IMRT to a median 60 Gy (range, 39.9–69.0 Gy). The majority of patients received chemotherapy with radiation The Kaplan-Meier estimate of overall survival (OS) at 1 year for definitively irradiated patients was 29%. Five patients without distant metastases had no evidence of disease at last followup. Use of IMRT versus 3DRT did not influence toxicity [71]. Hyperfractionated radiation regimens delivering a total of around 46 Gy have been most effective both with and without doxorubicin [12, 40, 72]. Wong and coworkers also noted skin changes, esophageal toxicity, and radiation myelopathy [59]. 

## 9. Chemotherapy

It has been found that *in vitro*, anaplastic cell lines express less multiple drug resistance (mdr1) mRNA and P-glycoprotein while expressing more multidrug resistance–associated protein (MRP) [73, 74]. This could explain the almost uniformly poor outcomes with chemotherapy. Doxorubicin is the most common agent used. A literature review by Ahuja showed a response rate around 22% for doxorubicin [75]. Swaak-Kragten and others did a retrospective analysis of seventy-five ATC-patients treated between 1972 and 2003 in the Netherlands. Thirty-six patients underwent up-front surgery of which with 53% had a R0(negative microscopic resection margin)/R1(positive microscopic resection margin) resection. Prior to 1988, adjuvant treatment consisted of conventional radiotherapy (RT) and/or chemotherapy (CT). After 1988, 30 eligible patients were enrolled in a protocol consisting of locoregional radiotherapy in 46 fractions of 1.1 Gy, given twice daily, followed by prophylactic irradiation of the lungs (PLI) in 5 daily fractions of 1.5 Gy. During radiation, low-dose doxorubicin (15 mg/m^2^) was administered weekly followed by adjuvant doxorubicin (50 mg/m^2^) 3-weekly up to a cumulative dose of 550 mg/m^2^. Twenty-five ineligible patients were treated conventionally. The overall median survival was 3 months, 1-year OS 9%. Locoregional control was significantly higher in patients who had undergone R0/R1 resection or chemoradiation, with best results for patients who underwent both (complete remission in 89%). However, the survival benefit of patients who reached CR remained borderline (median OS 7 months, 1-year OS 32%). The three patients who survived for more than 5 years had undergone R0/R1 surgical resection and chemoradiation. Acute toxicity in the protocol group was significantly higher than that in the nonprotocol group, with 46% versus 11% grade 3 pharyngeal and/or esophageal toxicity [30]. 

 In a study from Serbia, between 1997 and 2007, 16 inoperable patients were treated with radiotherapy at 60 Gy followed by doxorubicin 60 mg/m^2^ and cisplatin 40 mg/m^2^ every 3 weeks. The overall response rate (ORR) was 25% (95% CI: 7–55). No toxic deaths occurred or grade 4 adverse events were reported after radiotherapy. Grade 4 toxicity was seen in 3 patients after chemotherapy. Mean patient OS was 12.33 months (95% CI: 9.09–15.56) and median OS 11.0 months (95% CI: 8.56–13.44) [31]. Shimaoka and colleagues reported three complete and three partial responders out of 19 patients treated with doxorubicin and cisplatin [76]. Valproic acid, a histone deacetylase inhibitor, enhanced the effect of doxorubicin on anaplastic thyroid cancer cell lines in a preclinical study [77]. 

Paclitaxel was found to have a response rate of 53% (95% confidence interval: 29–76%) with one complete response and nine partial responses in 19 evaluable patients in a study conducted by Ain and colleagues [69]. A nonconventional response rate definition was utilized in this study. A recent study done in Japan compared overall survival after induction chemotherapy by weekly paclitaxel administration for patients with stage IVB (nine patients) and IVC (four patients) disease with that of ATC patients with stage IVB (*n* = 50) and IVC (*n* = 13) treated without paclitaxel. Complete response was seen in one, and two demonstrated partial response in the stage IVB group and one patient showed PR in stage IVC. After paclitaxel, curative surgery and adjuvant therapy were performed for four patients with stage IVB. All four patients were reported to be alive and disease-free 32 months after treatment. All four patients with stage IVC died of carcinoma within 8 months. Overall survival of stage IVB patients with induction chemotherapy was better (*P* = .0213) than that without the chemotherapy and also better (*P* = .0467) than those with chemotherapy other than paclitaxel. However, induction chemotherapy did not improve the overall survival of stage IVC patients (*P* = .2002) [78]. Docetaxel was administered intravenously at a dose of 60 mg/m^2^ over the course of 1 h every 3 weeks in seven patients with ATC who had received no prior chemotherapy in recent single center study in Japan. One patient had complete response, two patients had stable disease, and four patients had progressive disease. The response rate was 14%, and the disease control rate (complete/partial response plus stable disease) was 43%. The median time to progression was 6 weeks (range, 1–50). The authors report that the toxicity was tolerable [79]. In a small prospective study of 6 patients from Austria, standard external beam radiation of 60 gy was combined along with docetaxel at 100 mg fixed dose every 3 weeks for a total of six cycles starting within the first week of radiation. One patient had only completed radiation at the time of the report. Four patients achieved complete remission and two partial response. After a median followup of 21.5 months (range, 2–40 months), five patients were alive [62]. Paclitaxel when combined with manumycin showed the most response in combination when compared to either of the agents alone in anaplastic thyroid cancer cell lines in nude mouse [80]. In preclinical studies paclitaxel, gemcitabine, and vinorelbine have showed activity against ATC cell lines [81]. 

Chemotherapies with bleomycin, etoposide, cisplatin, and methotrexate have poor response rates with no long-term survival [9, 65]. Other combinations with vincristine and melphalan have not produced any improvements in the rate of responders [75, 82]. 

## 10. Emerging Therapies

As more data becomes available regarding the molecular pathogenesis of ATC, more targeted therapies are appearing in the clinic. Two of the most promising class of agents are the small-molecule tyrosine-kinase angiogenesis inhibitors and vascular disrupting agents. There are two small-molecule tyrosine-kinase inhibitors in the midst of Phase II clinical trials including imatinib mesylate (Gleevac, Novartis, East Hanover, NJ, USA) and sorafenib (Nexavar, Onyx, San Fransisco, Calif, USA). Imatinib mesylate is an orally available selective c-abl tyrosine-kinase inhibitor. It has been shown effective *in vitro* in anaplastic thyroid cell lines [93, 94]. Another study did not find it effective [95]. Sorafenib is a novel small-molecule tyrosine-kinase inhibitor which acts on the raf-1 serene/threonine kinase. BRAF mutations are thought to be an important event in the evolution of ATC and are a potential therapeutic target for treatment. Sorafenib also blocks the receptor tyrosine kinases to the vascular endothelial growth factor receptor 2 (VEGFR2) and platelet-derived growth factor receptor *β* (PDGFR-*β*) and thus has antiangiogenesis properties as well. Sorafenib inhibited the growth of rat orthotopic ATC xenografts, and the survival of test animals was improved in recently reported preclinical study [96]. This agent was studied in a clinical trial by Gupta-Abramson and colleagues in patients with advanced thyroid cancer, regardless of histology. Two of the 30 patients studied had anaplastic thyroid cancer. Both patients progressed despite therapy. However, one patient had a 50% decrease in size of a shoulder nodule at 4 weeks of treatment, before progressing with pericardial nodules at 7 weeks [97]. Sorafenib, at 400 mg bid on a 28 day cycle, was used on 16 patients with ATC, who had failed chemotherapy and/or radiotherapy. Median age was 55 years, two of the 15 (13%) evaluable patients had partial response, and 4 (23%) had stable disease. Median time in study was 2 months. Median duration of PD/SD was 5.1 months, and median duration of survival was 3.5 months. This study is ongoing at this time [64]. 

Axitinib (AG-013736) is an oral, selective inhibitor of VEGFRs 1, 2, and 3, and preclinical studies show that it blocks angiogenesis and tumor blood flow in preclinical models [98]. Cohen and colleagues studied this agent in a clinical trial on patients with advanced thyroid cancer. Two of the 60 patients had ATC. One patient has a partial response, and the other one progressed in spite of treatment [99]. 

Fosbretabulin is a derived from the African bush willow, *Combretum caffrum*. It is a novel tubulin-binding, vascular=disrupting agent and should be differentiated from the angiogenesis inhibitors discussed above. It displays potent and selective toxicity towards tumor vasculature and is thought to act by endothelial disruption of established tumor vasculature [100, 101]. The agent binds avidly to tubulin at the colchicine-binding site to inhibit microtubule assembly and destabilizes the cytoskeleton [102]. Fosbretabulin has also been shown to enhance or act synergistically with radiation and several chemotherapeutic agents [103, 104]. In a Phase II study in 26 patients with ATC, this agent was found to be well tolerated with grade 3 or greater toxicity being observed in 35% of patients. Median survival was 4.7 months, with 34% and 23% alive at 6 and 12 months, respectively. Median duration of stable disease was 12.3 months (range 4.4–37.9). Low-baseline soluble intracellular adhesion molecule-1 (sICAM) appeared to predict better event-free survival [63]. In a Phase 2/3 trial, fosbretabulin, paclitaxel, and carboplatin combination was compared to carboplatin and paclitaxel only in 80 patients with ATC. Interim results have been presented at the European Society of Medical Oncology meeting in Milan, Italy (October 2010). Preliminary results showed that the combination is well tolerated and showed an improvement in overall survival from 4.1 months to 5.1 months, with hazard ration of 0.71. *P* value could not be calculated, as the study had to be truncated due to poor accrual. One-year survival was almost doubled with fosbretabulin, when compared to chemotherapy alone (23% versus 9%). OS was objectively longer in patients less than 60 years of age, increasing from a median of 3.1 months to 10.9 months (HR of 0.38, 95% CI: 0.16, 0.88) [61]. 

In a pilot study, two patients with ATC had intra-tumor injection of retroviral vector with the human IL-2 gene and the suicide gene thymidine kinase of HSV type 2. Gene therapy resulted in marked increase in *T*-helper-type 1 cytokine profile and induced radiologically proven necrosis in the tumor [105]. Sodium stibogluconate is a trivalent antimonial compound, traditionally used for the treatment of leishmaniasis. It has recently been shown as an inhibitor of selective protein tyrosine phosphatases and could augment immune system activation when combined with interferon alfa-2b [106]. The MMP-activated anthrax lethal toxin (LeTx) was shown to inhibit orthotopic ATC xenograft progression in both toxin-sensitive and toxin-resistant ATC cells via reduced endothelial cell recruitment and subsequent tumor vascularization [92]. Other agents that have been reported to be effective *in vitro* studies include replication-competent vaccinia virus (GLV-1h68) [107]. Agents that are in the midst of preclinical evaluation are listed in Table 3.

## 11. Summary and Conclusions

The overall prognosis of ATC continues to be poor, with the 5-year survival in recent SEER database study to increase the order of 5.6% to 11.4% among all regions of the United States [108]. There has not been much improvement in response rates achieved above the 20% response rate seen with doxorubicin over the years. Further research is needed to evaluate new treatments for this almost uniformly fatal disease.

## Figures and Tables

**Table 1:** Chart review and retrospective studies.

Authors	Year	Site	No. of pts	Study details	Results
Swaak-Kragten et al. [30]	2009	Netherlands	75	Chart Review 1972–2003	When treated with Doxorubicin and radiation, the median survival was 3 months. 1 yr OS was 9%. Locoregional control was significantly higher in patients who had undergone R0/R1 resection or chemoradiation, with best results for patients who underwent both (complete remission in 89%).

Vrbic et al. [31]	2009	Serbia	16	Chart Review 1997–2007	Radiation was combined with doxorubicin 60 mg/m^2^ and cisplatin 40 mg/m^2^ every 3 weeks. Overall response rate was of 25% (95% CI: 7–55). Mean patient OS was 12.33 months (95% CI: 9.09–15.56) and median OS 11.0 months (95% CI: 8.56–13.44).

Yau et al. [32]	2008	Hong Kong	50	Chart review 1996–2006	Median survival was 97 days. On univariate analysis, age ≤ 65 (*P* ≤ .01), absence of metastatic disease at presentation (*P* < .01), surgical resection (*P* < .01), and postoperative radiotherapy was associated with longer survival. Cytotoxic chemotherapy was not associated with longer survival.

Lim et al. [33]	2007	USA	37	Chart review 1984–2006	A median radiation of dose 5760 cGy, >4500 cGy in 32 (87%) was administered through hyperfractionated or once-daily schedules. Median number of treatments received 6, >4 in 24 (65%). 2-year outcomes: locoregional control 25%; progression free survival 8%; overall survival 18%. 6 patients remained alive at the time of last followup with survival durations of 4, 11, 12, 57, 59, and 141 months, respectively.

Lee et al. [34]	2006	Korea	15	Chart review 1988–2003	The mean overall survival time of the 15 patients was 237 days (range, 28–717 days). The 6, 12, 18, and 24-month survival rates were 33%, 26%, 13%, and 0%.

Wang et al. [35]	2006	Canada	47	Chart review 1983–2004	The 6-month local progression-free success rate was 95% (*P* = .0001) with radical radiotherapy > 40 Gy compared to 64% with palliative radiotherapy at <40 Gy. Survival was 11 months with radical and 3 months with palliative (*P* = .0001). The median overall survival time in patients with twice-daily fractionation (13 months) was 3 months longer than patients treated with once-daily fractionation (10 months), but the difference was not statistically significant (*P* = .3).

Veness et al. [36]	2004	Australia	18	Chart review 1979–2002	Median survival was 6.2 months.

Haigh et al. [6]	2001	Canada	33	Chart review	In patients treated with potentially curative resection, median survival was 43 months and was 3 months with palliative resection (*P* = .002). The median survival of 3.3 months with only chemotherapy and irradiation and palliative resection (*P* = .63).

McIver et al. [27]	2001	USA	134	Chart review 1949–1999	Extent of resection or completeness of resection did not affect survival. (*P* > .4). Postoperative radiotherapy improved median survival (5 versus 3 months) but was not significant (*P* < .08).

Besic et al. [37]	2001	Slovenia	162	Chart review 1972–1998	82 patients with distant metastasis at presentation were excluded. Patients were divided into primary surgery (*n* = 26) or primary chemotherapy and/or radiation therapy. One-year survival was similar in both groups (*P* = .17).

Sugino et al. [38]	2002	Japan	40	Chart review 1989–1999	The one-year survival rate for surgery was 60%. The survival rate without surgery was 21%. Surgery and chemotherapy were both used in some patients. One-year survival rates for patients with small focus of anaplastic thyroid cancer with well-differentiated thyroid cancer were 73%.
Heron et al. [39]	2002	USA	32	Chart review 1952–1999	Patients were divided into two groups. Group 1 patients between 1952–80 (*n* = 9) and Group 2 between 1981–1999 (*n* = 23). Group 1 patients received once daily radiation and Group 2 patients twice-daily radiation with chemotherapy of doxorubicin, pacitaxel, vincristine, or cisplatin There was a combination of therapies in both groups, with the above generalizations. 2-year survival was 44% in group 1 and 52% in group 2. Progression-free survival was 53% group 1 and 38% group 2. The authors concluded that hyperfractionated radiation with chemotherapy is associated with better survival but not progression-free survival.

Nilsson et al. [18]	1998	Sweden	81	Chart review 1971–1997	Eight patients (10%) survived more than 2 years and were treated with combinations of chemotherapy, radiotherapy, and surgery.

Tennevall et al. [40]	1994	Sweden	33	Chart review 1984–1992	Combination of hyperfractionated radiotherapy, doxorubicin, and debulking surgery. Preoperative radiation up to 30 Gy and post operative radiation up to 46 Gy. 20 mg doxorubicin per week. 48% had no local recurrence and, 24% died due to local failure. In 4 patients survival exceeded 2 yrs. Local control better with accelerated radiation therapy.

Venkatesh et al. [24]	1990	USA	121	Chart review	Median survival was 7.2 ± 10 months. 35% patients had well-differentiated thyroid cancer as well.

Junor et al. [10]	1992	UK	91	Chart review 1961–1986	Surgery and radiation were used. Total or partial thyroidectomy increased survival. 80% responded to radiation therapy.

Levendag et al. [41]	1993	Holland	51	Chart review 1970–1986	Local control achieved a median survival of 7.5 months and local residual disease 1.6 months.

Kim and Leeper [12]	1987	USA	41	Chart review 1979–1986	Only reporting Group 2 with anaplastic thyroid cancer. Weekly doxorubicin 10 mg/m^2^ prior to hyperfractionated radiotherapy with 160 cGy twice daily to total 57–60 cGy in 40 days. Initial complete remission rate was 84%. Local tumor control at 2 years was 68% with combined therapy. Median survival was 1 year.

**Table 2:** Prospective studies.

Author	Year	Site	No. of pts	Study details	Results
Sosa et al. [61]	2010	International	80	55 pts were randomized to paclitaxel/carboplatin and fosbretabulin, and 25 patients were randomized to receive paclitaxel and carboplatin only. Pts were followed until they died.	Fosbretabulin was well tolerated with carboplatin and paclitaxel. Improved overall survival (OS) in ATC from 4.1 months to 5.1 months. OS was longer in younger patients <60 yrs increasing from medial of 3.1 months to 10.9 months (HR: 0.38, 95% CI: 0.16, 0.88, *P* = .0222).

Troch et al. [62]	2010	Austria	6	Standard external beam radiation of 60 gy was combined along with docetaxel at 100 mg fixed dose every 3 wks for a total of six cycles starting within the first week of radiation.	One patient had only completed radiation at the time of the report. Four patients achieved complete remission, and two achieved partial response. After a median followup of 21.5 months (range, 2–40 months), five patients were alive.

Mooney et al. [63]	2009	USA	26	26 patients with biopsy-proven ATC received fosbretabulin at 45 mg/m^2^.	There was no objective response. Median survival was 4.7 months with 34% and 23% alive at 6 and 12 months, respectively. Median duration of stable disease in seven patients was 12.3 months (range, 4.4–37.9 months). Lower baseline sICAM-1 levels correlated with better event-free survival. Fosbretabulin was well tolerated with grade 3 toxicity in 34% and grade 4 in 4% of patients.

Nagaiah et al. [64]	2009	USA	16	Patients with biopsy-proven ATC who had progressed on cytotoxic chemotherapy with or without radiation were treated with sorafenib 400 mg BID on a 28 day cycle.	2 of the 15 evaluable patients (13%) had partial response, and 4 patients (27%) had stable disease. Median time in study was 2 months. Median duration of PD/SD was 5.1 months, and median duration of survival was 3.5 months.

Koussis et al. [65]	2006	Italy	56	Patients were divided into 3 groups. Group A: 19 patients radiotherapy, total thyroidectomy, and chemotherapy. Cisplatin once a week and by radiation at 36 Gy in 18 fractions over 3 weeks, followed by total thyroidectomy and by further chemotherapy with doxorubicin and bleomycin. Additionally, five patients received weekly docetaxel. Group B: consisted of 19 patients with distant metastasis at diagnosis who received chemotherapy (Platinum-based combination). Group C: consisted of 18 elderly patients in poor general condition; 6 received local radiation, while 12 did not receive any treatment.	Five complete responses were seen in patients from Group A. Four patients had long-term survival (14, 15, 24, and 41 months) with a disease-free survival interval of 6, 8, 11, and 32 months. Median survival rates for Groups A, B, and C was 12, 5.7, and 4 months, respectively.

Wallin et al. [66]	2004	Sweden	22	Hyperfractionated radiotherapy 1.6 Gy × 2 to a total target dose of 46 Gy given preoperatively, 20 mg doxorubicin was administered intravenously once weekly and surgery was carried out 2-3 weeks after the radiotherapy.	17 of these 22 patients were operated. Partial regression in 7 others; the one patient whose tumor failed to respond was treated only once daily. Two patients died of spinal cord necrosis and a third of pneumonitis due to the unexpected increase in radiation toxicity caused by the concurrent administration of doxorubicin. None of these 17 patients got a local recurrence. No survival data.

De Crevoisier et al. [67]	2004	France	30	Hyperfractionated accelerated radiotherapy and total of 6 cycles of doxorubicin/cisplatin was used.	Complete local response was seen in 19 patients. Overall survival at 3 years was 27% and median survival was 10 months. Death was related to local progression in 5% of patients.
Mitchell et al. [68]	2002	USA	28	28 patients with ATC without distant metastases received radiotherapy to the primary tumor and bilateral neck in 1.6 Gy fractions twice daily and 3 days per week, with concurrent doxorubicin 10 mg/m^2^ weekly. Three histological subsets: anaplastic carcinoma with giant and/or spindle cell features (*n* = 12); anaplastic carcinoma arising from papillary or follicular carcinoma (*n* = 8); and undifferentiated (*n* = 8).	The 3-year actuarial local control, metastasis-free survival, and overall survival rates were 47%, 8%, and 14%, respectively. Followup among the five currently living patients is 82, 27, 4, 3, and 1 months, respectively. Site of first failure was distant in 13 patients and local in 7 patients.

Ain et al. [69]	2000	USA	20	Patients received 96-hour continuous infusion of paclitaxel every 3 weeks for 1 to 6 cycles; the first 7 patients received 120 mg/m^2^ per 96 hours, and the rest received 140 mg/m^2^ per 96 hours.	Of the 19 evaluable patients, there was a 53% total response rate (95% confidence interval; 29–76%) including 1 complete response and 9 partial responses (including one off protocol). Nonconventional response criteria.

Busnardo et al. [70]	2000	Italy	39	A total of 16 patients (Group 1) were treated with total thyroidectomy, radiation therapy, and chemotherapy in various orders. Nine patients with distant metastases at diagnosis (Group 2) received chemotherapy; one patient had disappearance of lung metastases and was then treated by total thyroidectomy and further chemotherapy. Group 3 consisted of 14 elderly patients in poor general conditions; 4 of these received local radiation therapy, while the remaining did not receive any treatment.	Median survival rate was 11 month for Group 1. It was 5.7 months for Group 2, and 4 months for Group 3. Multimodality treatment was associated with increased survival. Nine out of 16 patients, who underwent surgery and complementary treatment, had no local progression.

Mitchel et al. [16]	1999	UK	17	Twice-daily radiation for 5 days a week to a total dose of 60.8 Gy in 32 fractions over 20–24 days was given in two or three phases.	Three patients with ATC demonstrated a complete clinical response, and 7 patients achieved a partial response. Five patients had stable disease, and 2 patients died before radiotherapy was completed.

Schlumberger et al. [19]	1991	France	20	Chemotherapy and radiation for patients aging less than 65 years treated with doxorubicin and cisplatin; patients older than 65 years with mitoxantrone and radiation at 17.5 Gy.	Three patients survived more than 20 months; 5 patients had complete local tumor response.

Tennevall et al. [60]	1990	Sweden	16	Hyperfractionated radiotherapy, doxorubicin, and debulking surgery. The radiotherapy was preoperatively administered to a target dose of 30 Gy in 3 weeks, and postoperatively to an additional dose of 16 Gy in 1.5 weeks. 20 mg doxorubicin was used.	Five patients achieved local complete remission, and 3 patients were alive disease-free at 10, 30, and 30 months, respectively, after diagnosis. Only 6 patients succumbed to local failure.

Kim and Leeper [12]	1987	USA	19	Group 2 patients with anaplastic giant and spindle cell carcinoma of the thyroid (*n* = 19) received doxorubicin (10 mg/m^2^) before hyperfractionated radiation. Radiation therapy was carried out with a fractional dose of 160 cGy per treatment twice a day for 3 days per week to a total dose of 5760 cGy in 40 days.	Local tumor control rates at 2 years after combined therapy were 77% and 68%, respectively. The median survival time was 4 years for group 1 and 1 year for group 2.

**Table 3:** Preclinical agents.

Mechanism	Agents	Studies
Loss of p53 or abnormal p53 is expressed in anaplastic thyroid cancer [83]	Adenovirus with wild type p53	Blagosklonny et al. showed that anaplastic thyroid cancer cell lines infected with the p53 adenovirus became more sensitive to doxorubicin.
Unknown [84]	Bovine seminal ribonuclease	*In vivo* tumor regression of anaplastic thyroid cancer.
Inhibition of cyclin-dependent kinase activity [85]	Bone morphogenic protein	Inhibition of 4 of 6 anaplastic thyroid cancer cell lines.
EGFR Tyrosine-kinase inhibitor [86]	ZD1839 (gefitinib)	EGFR is overexpressed in anaplastic thyroid cancer *in vitro/vivo* and gefitinib induces apoptosis *in vitro* and inhibits subcutaneous mouse models of anaplastic thyroid cancer.
EGFR Monoclonal antibody [87]	Cetuximab	As single agent cetuximab had no activity, but with irinotecan it inhibited orthotopic anaplastic thyroid cancer xenografts more than doxorubicin.
EGRF/VEGF Receptor blocker [88]	AEE788	*In vitro* inhibition and nude-mouse inhibition with pacitaxel.
Histone Deacetylase Inhibitors [77, 89–91]	Valproic acid and other novel agents	Restored radio iodide uptake and restoration of p53 or pseudo- p53 activity.
Inhibition of gelatinase class of matrix metalloproteinases (MMP) that are activated in ATC [92]	MMP-activated LeTx	Reduced endothelial cell recruitment and subsequent tumor vascularization

## Abbreviations

ATC: Anaplastic thyroid cancer
CA4P: Combretastatin A4 phosphate, also known as fosbretabulin.
